# Automated patient-robot assignment for a robotic rehabilitation gym: a simplified simulation model

**DOI:** 10.1186/s12984-022-01105-4

**Published:** 2022-11-16

**Authors:** Benjamin A. Miller, Bikranta Adhikari, Chao Jiang, Vesna D. Novak

**Affiliations:** 1 Department of Electrical and Computer Engineering, University of Wyoming, 1000 E University Ave., Laramie, WY 82071 USA; 2 Department of Electrical Engineering and Computer Science, University of Cincinnati, 2600 Clifton Ave., Cincinnati, OH 45221 USA

**Keywords:** Rehabilitation robotics, Rehabilitation gym, Group rehabilitation, Optimization, Mathematical modeling, Task scheduling, Mixed-integer nonlinear programming

## Abstract

**Background:**

A robotic rehabilitation gym can be defined as multiple patients training with multiple robots or passive sensorized devices in a group setting. Recent work with such gyms has shown positive rehabilitation outcomes; furthermore, such gyms allow a single therapist to supervise more than one patient, increasing cost-effectiveness. To allow more effective multipatient supervision in future robotic rehabilitation gyms, we propose an automated system that could dynamically assign patients to different robots within a session in order to optimize rehabilitation outcome.

**Methods:**

As a first step toward implementing a practical patient-robot assignment system, we present a simplified mathematical model of a robotic rehabilitation gym. Mixed-integer nonlinear programming algorithms are used to find effective assignment and training solutions for multiple evaluation scenarios involving different numbers of patients and robots (5 patients and 5 robots, 6 patients and 5 robots, 5 patients and 7 robots), different training durations (7 or 12 time steps) and different complexity levels (whether different patients have different skill acquisition curves, whether robots have exit times associated with them). In all cases, the goal is to maximize total skill gain across all patients and skills within a session.

**Results:**

Analyses of variance across different scenarios show that disjunctive and time-indexed optimization models significantly outperform two baseline schedules: staying on one robot throughout a session and switching robots halfway through a session. The disjunctive model results in higher skill gain than the time-indexed model in the given scenarios, and the optimization duration increases as the number of patients, robots and time steps increases. Additionally, we discuss how different model simplifications (e.g., perfectly known and predictable patient skill level) could be addressed in the future and how such software may eventually be used in practice.

**Conclusions:**

Though it involves unrealistically simple scenarios, our study shows that intelligently moving patients between different rehabilitation robots can improve overall skill acquisition in a multi-patient multi-robot environment. While robotic rehabilitation gyms are not yet commonplace in clinical practice, prototypes of them already exist, and our study presents a way to use intelligent decision support to potentially enable more efficient delivery of technologically aided rehabilitation.

## Background

### Rehabilitation robotics and the robotic gym

Over the last decade, rehabilitation robots have demonstrated the ability to deliver motor rehabilitation with results comparable to a human therapist [1–3]. By physically guiding the patient’s limb and applying either assistive or challenging forces [4, 5], such robots can effectively reduce the physical workload of the human therapist. Traditionally, rehabilitation robots were operated in a setup consisting of one robot, one patient, and one supervising therapist [1, 2]; this was likely simply due to the high cost of individual robots, which made it difficult for most rehabilitation centers to own more than one robot. However, the last few years have seen a push toward more affordable rehabilitation robotics [6], which enables rehabilitation centers to own more than one robot and introduces opportunities for multi-robot setups.

The first steps beyond the classic “one patient, one robot, one therapist” model involved connecting two robots (or passive sensorized rehabilitation devices), allowing two patients to exercise together either independently or in a competitive/collaborative manner while supervised by a single therapist [7–12]. To reduce the burden on the therapist, such two-robot setups commonly include automated difficulty adaptation algorithms that aim to keep the exercise difficulty appropriate for both patients [13–15], removing the need for the therapist to constantly modify the exercise settings. Short-term studies have shown benefits to such paired rehabilitation such as improved motivation, exercise intensity and motor learning [7–12], and a recent clinical trial found greater improvements in functional outcome after paired therapy than after individual therapy [16].

As the intuitive next step beyond connecting two robots, rehabilitation centers may take the form of “robotic gyms” where multiple patients train with multiple robots or passive sensorized devices in a group setting. Such a robotic gym was first demonstrated with six rehabilitation devices as early as 2016, and a pilot clinical trial suggested that it may be more efficient than the traditional “one patient, one robot, one therapist” model since it may allow therapists to supervise multiple patients simultaneously [17]. In the last few years, several rehabilitation centers have set up robotic gyms with multiple robots: for example, Fondazione Don Carlo Gnocchi, Italy [18], Shirley Ryan AbilityLab, USA; SRH Gesundheitszentrum Bad Wimpfen, Germany. Additionally, a 2020 multicenter clinical trial was conducted with such a robotic gym: patients alternated between four cheaper lower-cost rehabilitation devices, with results comparable to those previously seen with larger robots [3]. In this four-device study, each therapist was assigned to supervise 3 patients, and the authors again emphasized that such group settings may allow new organizational models that are more cost-efficient than the traditional “one patient, one robot, one therapist” model [3]. An observational study by the same team suggested that a therapist may be able to effectively supervise up to four patients exercising with four robots [19].

### Improving therapist support in the robotic gym

While having a single therapist supervise multiple patients in a robotic gym may be cost-effective, there is a risk of divided attention: a therapist may not be able to effectively monitor all the patients, leading to suboptimal therapy. For example, if a device trains elbow flexion/extension, a patient’s elbow may become fatigued and the patient may benefit to moving to a different device that trains finger function instead; however, if the therapist does not notice this, the patient may continue exercising with the elbow device and getting progressively more tired and frustrated. As a more extreme example, if a robot is not optimally physically aligned to the patient and the therapist does not notice this, there is a risk of patient injury [20]. However, we believe that such issues could be avoided and cost-effectiveness in a robotic gym could be improved further if the therapist is provided with effective software support: a central system that collects information from all robots in the gym and presents it to the therapist.

Rehabilitation robots are already able to assess patient task performance and motor function using built-in sensors [21–24]. In a robotic gym, each robot could independently monitor the current patient, and the information from all patients could be aggregated and presented to a supervising therapist via a central portal, thus reducing therapist workload. Initial steps have already been taken in this direction: for example, Hocoma AG (Switzerland), a major manufacturer of rehabilitation robots, has introduced the Hoconet software portal, which allows a therapist to create a patient database and gather data from multiple robots in a centralized fashion.

To further reduce therapist workload and potentially improve group rehabilitation outcome, we could endow the robotic gym with a software agent that would monitor the patients as a group. The agent could then estimate when a patient might benefit from moving to a different robot (due to, e.g., fatigue, boredom, or simply lack of improvement in the current exercise). It could then suggest such a change to the patient(s) or supervising therapist, who could either accept or reject it. In the long term, this may become a bidirectional exchange of information between the therapist and the robotic supervision system: the robot intelligence could study when the therapist chooses to accept or reject its suggestions, and could learn to adapt its suggestions accordingly. More extremely, such centralized planning by an artificial intelligence could even be used in situations where a therapist is not available: for example, during a weekend group session supervised by a technician or nurse rather than a therapist. Again, basic steps have been taken in this direction: for example, Hocoma AG has introduced the Extra Time software, which allows an aide to run previously described exercises when a therapist is not available. However, the Extra Time software does not perform any monitoring or planning.

Such a centralized patient monitoring and patient-robot assignment planning system has not been implemented in existing robotic gyms, where patients either do not switch between devices within the session [3] or switch between them arbitrarily after a predefined time period [6]. However, it should be possible: theoretical models suggest that, given knowledge about ability levels, it is possible to design a multi-task training regimen that maximizes long-term retention across different trained functions [25, 26], though this has not been applied to robotics.

## Contribution of current paper

In this paper, we introduce the concept of an intelligent patient-robot assignment system for a robotic gym that monitors all patients’ exercise performance and dynamically assigns them to available robots in order to optimize training outcome for the entire patient group. Realistically, this is a very complex problem involving numerous uncertainties (e.g., the difficulty of estimating actual patient motor ability from performance in a single specific task [21–24]), and the success of a specific solution could only be determined via long-term human subjects testing with actual people with motor impairments. However, as initial steps toward a realistic system, we:

- Developed a simplified mathematical model for patient-robot assignment and training planning in a robotic gym,
- Adopted mixed-integer nonlinear programming algorithms to find optimal assignment and training solutions,
- Verified the proposed assignment solutions in different simulated situations and demonstrated effective training results.
- Discussed limitations of the model and next steps.

## Methods

This section is divided as follows. We first present the simplified robotic rehabilitation gym scenario in plain English (Scenario description) and using mathematical formulations (Mathematical scenario formulation). We then present the mixed-integer programming algorithms used for dynamic patient-robot assignment (Optimization algorithms) and multiple evaluation scenarios in which the performance of these algorithms was evaluated (Evaluation methodology).

### Scenario description

In our simplified scenario, the robotic rehabilitation gym consists of *M* patients exercising with *N* robots for a period of *G* time steps. Each patient has *K* motor skills that they need to improve; these can be considered to be, for example, different upper limb and lower limb abilities as separated by clinical scales such as the Motor Assessment Scale [27] or the Fugl-Meyer Assessment [28], but are kept general for purposes of the simplified scenario. Similarly, a robot could also be a passive sensorized rehabilitation device but is kept as a general ‘robot’ for purposes of our scenario.

For the current paper, multiple simplifications and constraints of the scenario were implemented. The scope of these simplifications and ways in which they could be expanded are examined further in the Discussion. First, there were several constraints on robot use that we consider reasonable:

- At any given time, only a single patient can use a given robot. While competitive and cooperative two-robot setups could be modeled as a single robot that can be used by two patients [7–12], such setups are currently a minority.
- At any given time, a given patient can only use a single robot. While there are some setups where a patient can train with two robots simultaneously (e.g., an arm and leg robot [29]), they are relatively rare.
- Each robot has an associated nonnegative time required to ‘exit’ the robot after training that is constant across patients and does not contribute to skill improvement. Realistically, there is both an ‘enter’ and an ‘exit’ time for each robot, which represents the time needed to, e.g., adjust the lengths of the robot’s segments to the patient, strap the patient to the robot before exercise, and unstrap them after exercise. The time may in practice be different for different patients and may vary depending on the temporal sequence of patients (e.g., if a patient is followed by a patient of roughly the same size, reducing readjustment time). However, we consider simplifying this as an overall ‘exit’ time to be reasonable for the current study.
- As a result of the above exit time, a patient cannot enter a new robot until they have exited their current robot, and a patient cannot enter a robot until the previous patient has exited that robot.

Second, there were two constraints on training schedules that we also consider relatively reasonable:

- All patients begin and finish training simultaneously; none can train before the starting time or after the end time. In practice, patients may arrive and leave one by one.
- A patient may only train with a given robot once for a single uninterrupted period. For example, they cannot leave the robot, train with another robot, and come back to the first robot; as a second example, they cannot train with the robot, take a break where they do nothing, and continue training with the same robot. This is likely reasonable if the time period to be modeled is a single session, and can be expanded later.

Third, there were five simplifications related to skill acquisition, of which the last two are quite major and are discussed extensively in the Discussion:

- Each robot only trains a single motor skill. Larger robots can train multiple skills simultaneously (e.g., both distal and proximal upper extremity function in the ARMin [2]), and there is evidence that training one skill generalizes to improvements in other skills (e.g., distal to proximal [30]), but we consider this a reasonable initial simplification that can easily be expanded later.
- Each skill is only trained by a single robot. In practice, a robotic gym may have multiple robots that all train the same skill (e.g., multiple identical robots), but we again consider this a reasonable initial simplification that can easily be expanded later.
- Once a patient is assigned to a robot, no further choices need to be made for that robot. In practice, rehabilitation robots have adjustable difficulty settings and control strategies [5] that are set either manually by the therapist or automatically by an intelligent algorithm.
- Each skill improves as a deterministic function of time spent training on a robot that trains that skill, and depends on no other factors. This is a strong simplification; while such learning functions have been described in the literature [31, 32], they are not deterministic, and improvement is influenced by numerous other factors (e.g., forgetting [25]).
- Each patient’s current skill levels are available to the patient-robot assignment algorithms at all times, and the functions that relate improvement to training time are also known. This is a very strong simplification: while rehabilitation robots can assess patient motor function using built-in sensors, this estimate is not completely accurate [21–24]; furthermore, standardized clinical tests of motor function such as the Fugl-Meyer Assessment [28] are not perfectly accurate and cannot be conducted during a rehabilitation session since they require significant time. Similarly, the learning function is not known to rehabilitation robots and can only be imperfectly estimated.

### Mathematical scenario formulation

For the scenario described in the previous subsection, we define a set of rehabilitation robots, $R:=\{{r}_{1},\dots ,{r}_{N}\}$, a group of patients, $P:=\{{p}_{1},\dots ,{p}_{M}\}$, and a set of motor skills, $S:=\left\{{s}_{1},\dots ,{s}_{K}\right\}$, to be trained for each patient. Let $N$ be the number of robots, $M$ be the number of patients and $K$ be the number of skills. To follow the simplifications above, $N = K$ and each skill is trained by exactly one robot. The training session consists of $G$ discrete time steps, i.e., $G$ is the final time training can be done. We also define $H$ as the final time a patient may start training on a new robot. Both $G$ and $H$ are non-negative integers, and $G\ge H$. In our specific case, we set $H = G$, allowing a patient to start training on a robot at the last time step and train for a single time step. The time steps can be used to represent any amount of time as long as the duration of each time step is the same. For each robot ${r}_{i}\in R$, the robot’s exit time is defined as a non-negative integer ${e}_{{r}_{i}}$. When no time is needed to exit a robot, ${e}_{{r}_{i}}$ = 0. For each patient ${p}_{j}\in P$, we are given skill curves that determine skill improvements as a function of time spent training on different robots. This encapsulates the last two simplifications in the previous subsection. The objective of dynamic patient-robot assignment and training planning is to find a schedule of the patients’ skill training on the robots that optimizes the total skill gain across the patients.

The scheduling problem can be framed as a mixed-integer nonlinear programming (MINLP) problem. There are three basic MINLP formulations: the time-indexed formulation, the disjunctive formulation, and the rank-based formulation [33]. The choice of MINLP formulations is based on their flexibility to model a problem and the computational efficiency to solve the problem. We applied both time-indexed (Time-indexed model) and disjunctive (Disjunctive model) models to our scheduling problem. After presenting broad models, we applied several simplifications for the current study, described in “Simplifications for our study”.

#### Time-indexed model

In time-indexed models, a schedule is created by determining, for each time step $t\le H$, whether a patient starts training on a given robot and how long the patient trains on the robot. The decision variables used for the time-indexed model are defined as follows:

- ${x}_{{r}_{i},{p}_{j},t}$ is a Boolean variable that is equal to 1 if patient ${p}_{j}$ starts training on robot ${r}_{i}$ at time step $t$.
- ${d}_{{r}_{i},{p}_{j}}$ is a nonnegative integer that represents the amount of time patient ${p}_{j}$ trains on robot ${r}_{i}$.

Unlike the original time-indexed models for process engineering [34] where the duration of a task is known a priori, our model introduces the variable ${d}_{{r}_{i},{p}_{j}}$ to determine when a patient should stop training on a given robot as part of the decision-making. The time-indexed MINLP model is then formulated as maximizing objective function (1), subject to constraints (2)–(8):

1 $$\mathrm{max}\sum_{i=1}^{N}\sum_{j=1}^{M}\sum_{t=1}^{H}{f(x}_{{r}_{i},{p}_{j},t},{d}_{{r}_{i},{p}_{j}})$$

subject to

2 $${\sum }_{j=1}^{M}{x}_{{r}_{i},{p}_{j},t }\le 1, \forall {r}_{i}\in R, t\le H$$

3 $${\sum }_{i=1}^{N}{x}_{{r}_{i},{p}_{j},t }\le 1, \forall {p}_{j}\in P, t\le H$$

4 $$\sum_{i\in [a,b]}\sum_{t={s}_{{r}_{a},{p}_{j}}}^{{s}_{{r}_{a},{p}_{j}}+{d}_{{r}_{a},{p}_{j}}+{e}_{{r}_{a}}-1 }{x}_{{r}_{i},{p}_{j},t} \le 1, \, \forall \, {r}_{a},\,{r}_{b}\in R \text{ and } {r}_{a} \ne {r}_{b}, \, {p}_{j}\in P,$$

5 $$\sum_{t= {s}_{{r}_{i},{p}_{a}}}^{{s}_{{r}_{i},{p}_{a}}+{d}_{{r}_{i},{p}_{a}}+{e}_{{r}_{i}}-1}\sum_{j\in [a,b]}{x}_{{r}_{i},{p}_{j},t} \le 1,\, \forall \, {p}_{a},\,{p}_{b}\in P \text{ and } {p}_{a} \ne {p}_{b}, \,{r}_{i}\in R,$$

6 $${x}_{{r}_{i},{p}_{j},t}*t+{d}_{{r}_{i},{p}_{j}}-1\le G, \forall {r}_{i}\in R, {p}_{j}\in P, t\le H$$

7 $${\sum }_{i=1}^{N}\sum_{t={s}_{{r}_{a},{p}_{j}}}^{{s}_{{r}_{a},{p}_{j}}+{d}_{{r}_{a},{p}_{j}}+{e}_{{r}_a}-1}{x}_{{r}_{i},{p}_{j},t}= \sum_{t={s}_{{r}_{a},{p}_{j}}}^{{s}_{{r}_{a},{p}_{j}}+{d}_{{r}_{a},{p}_{j}}+{e}_{{r}_a}-1}{x}_{{r}_{a},{p}_{j},t}=1,\forall {r}_{i},{r}_{a}\in R, {p}_{j}\in P, t\le H$$

8 $$\sum_{t=1}^{ G}{x}_{{r}_{i},{p}_{j},t}\le 1, \forall {r}_{i}\in R, {p}_{j}\in P$$

The objective (1) maximizes the total skill gain across all patients during the training session, where

9 $$f({x}_{{r}_{i},{p}_{j},t},{d}_{{r}_{i},{p}_{j}})=\frac{{c}_{1,{r}_{i},{p}_{j}}*({c}_{2,{r}_{i},{p}_{j}} + {c}_{4,{r}_{i},{p}_{j}}*({d}_{{r}_{i},{p}_{j}}*{x}_{{r}_{i},{p}_{j},t}+{u}_{{r}_{i},{p}_{j}}))}{{c}_{2,{r}_{i},{p}_{j}}+{c}_{4,{r}_{i},{p}_{j}}*({d}_{{r}_{i},{p}_{j}}*{x}_{{r}_{i},{p}_{j},t}+{u}_{{r}_{i},{p}_{j}})+{c}_{3,{r}_{i},{p}_{j}}} -\frac{{c}_{1,{r}_{i},{p}_{j}}*({c}_{2,{r}_{i},{p}_{j}} + {c}_{4,{r}_{i},{p}_{j}}*{u}_{{r}_{i},{p}_{j}})}{{c}_{2,{r}_{i},{p}_{j}}+{c}_{4,{r}_{i},{p}_{j}}*{u}_{{r}_{i},{p}_{j}}+{c}_{3,{r}_{i},{p}_{j}}}$$

is the skill curve function with ${c}_{1,{r}_{i},{p}_{j}},{c}_{2,{r}_{i},{p}_{j}},{c}_{3,{r}_{i},{p}_{j}}$ determining the shape of the skill curve and ${c}_{4,{r}_{i},{p}_{j}}$ determining how far along the skill curve a patient advances per time step. Each $c$ value changes depending on the patient ${p}_{j}$ and robot ${r}_{i}$ pairing to create a custom skill curve. ${u}_{i,j}$ is the number of training time steps previously performed by patient ${p}_{j}$ with robot ${r}_{i}$ in previous sessions, plus one (with the plus one used to calibrate the function). The basic function is common to all patients and skills while the parameters may differ between patients and skills. It models a modified hyperbolic skill curve where patients tend to have rapid gains when first training a skill, then diminished returns as they train more [31, 32]. The $\frac{{c}_{1,{r}_{i},{p}_{j}}*({c}_{2,{r}_{i},{p}_{j}} + {c}_{4,{r}_{i},{p}_{j}}*{u}_{{r}_{i},{p}_{j}})}{{c}_{1,{r}_{i},{p}_{j}}+{c}_{4,{r}_{i},{p}_{j}}*{u}_{{r}_{i},{p}_{j}}+{c}_{3,{r}_{i},{p}_{j}}}$ portion of the equation represents the patient’s initial skill value before the current training session.

Constraints (2)–(5) are imposed on robot use. Constraint (2) ensures that only a single patient can start using a given robot at any given time. Constraint (3) ensures that a given patient can only start using a single robot at any given time. Constraint (4) ensures that a patient $${p}_{j}$$ cannot begin training on a new robot $${r}_{b}$$ until after they have exited their current robot $${r}_{a}$$ (i.e., $${s}_{{r}_{a},{p}_{j}}+{d}_{{r}_{a},{p}_{j}}+{e}_{{r}_{a} }-1$$, where $${s}_{{r}_{a},{p}_{j}}$$ is a nonnegative integer that represents the time when patient $${p}_{j}$$ starts training on robot $${r}_{a}).$$ This constraint is ignored if patient $${p}_{j}$$ does not train on robot $${r}_{a}$$ during the session. Constraint (5) ensures that a patient $${p}_{b}$$ cannot start training on a robot $${r}_{i}$$ until after the previous patient $${p}_{a}$$ on robot $${r}_{i}$$ has exited that robot and patient $${p}_{b}$$ has entered the robot (i.e., $${s}_{{r}_{i},{p}_{a}}+{d}_{{r}_{i},{p}_{a}}+{e}_{{r}_{i}}{p}_{a})$$. Again, this constraint is ignored if patient $${p}_{a}$$ does not train on robot $${r}_{i}$$ during the session. Constraint (6) ensures that no one can start training or continue to train after the final time step—i.e., no one can train at $$G+1$$. Constraint (7) ensures that a person can only train on a single robot at a time. Constraint (8) ensures that a patient may only train with a given robot once for a single uninterrupted period.

#### Disjunctive model

In disjunctive models, a schedule is created by determining what time a patient starts and finishes training on a given robot. The start and end times together implicitly capture the amount of time spent training on a robot. The decision variables used in the disjunctive model are defined as follows:

- ${x}_{{r}_{i},{p}_{j}}$ is the integer start time of patient $j$ on robot $i$.
- ${y}_{{r}_{i},{p}_{j}}$ is the integer end time of patient $j$ on robot $i$.
- ${a}_{{r}_{i},{p}_{a},{p}_{b}}$ is a Boolean precedence indicator that is equal to 1 if patient ${p}_{a}$ is on robot ${r}_{i}$ before patient ${p}_{b}$ and is equal to 0 otherwise.
- ${b}_{{r}_{a},{r}_{b},{p}_{j}}$ is a Boolean precedence indicator that is equal to 1 if patient ${p}_{j}$ is on robot ${r}_{a}$ before robot ${r}_{b}$ and is equal to 0 otherwise.
- ${z}_{{r}_{i},{p}_{j}}$ is a Boolean activation variable that is equal to 1 if patient ${p}_{j}$ uses robot ${r}_{i}$ during the training session and is equal to 0 otherwise.

The disjunctive MINLP model is formulated as maximizing objective (10), subject to constraints (11)–(15):

10 $$\mathrm{max}\sum_{i=1}^{N}\sum_{j=1}^{M}f({x}_{{r}_{i},{p}_{j}},{y}_{{r}_{i},{p}_{j}})$$

subject to

11 $$1\le {x}_{{r}_{i},{p}_{j}}\le {y}_{{r}_{i},{p}_{j}}\le G, \forall {r}_{i}\in R, {p}_{j}\in P$$

12 $$\left({x}_{{r}_{i},{p}_{a}}-({y}_{{r}_{i},{p}_{b}}+{e}_{i}-V*{a}_{{r}_{i},{p}_{a},{p}_{b}})\right)*{z}_{{r}_{i},{p}_{a}}*{z}_{{r}_{i},{p}_{b}}\ge 0, \forall {r}_{i}\in R, {p}_{a},{p}_{b}\in P$$

13 $$\left({x}_{{r}_{i},{p}_{b}}-({y}_{{r}_{i},{p}_{a}}+{e}_{{r}_{i}}-V*(1-{a}_{{r}_{i},{p}_{a},{p}_{b}}))\right)*{z}_{{r}_{i},{p}_{a}}*{z}_{{r}_{i},{p}_{b}}\ge 0, \forall {r}_{i}\in R,{p}_{a},{p}_{b}\in P$$

14 $$\left({x}_{{r}_{a},{p}_{j}}-({y}_{{r}_{b},{p}_{j}}+{e}_{{r}_{b}}-V*{b}_{{r}_{a},{r}_{b},{p}_{j}})\right)*{z}_{{r}_{a},{p}_{j}}*{z}_{{r}_{b},{p}_{j}}\ge 0, \forall {r}_{a},{r}_{b}\in R, {p}_{j}\in P$$

15 $$\left({x}_{{r}_{b},{p}_{j}}-({y}_{{r}_{a},{p}_{j}}+{e}_{{r}_{a}}-V*(1-{b}_{{r}_{a},{r}_{b},{p}_{j}}))\right)*{z}_{{r}_{a},{p}_{j}}*{z}_{{r}_{b},{p}_{j}}\ge 0, \forall {r}_{a},{r}_{b}\in R, {p}_{j}\in P$$

The objective (10) maximizes the total skill acquisition across all patients during the training session, where

16 $${f(x}_{i,j},{y}_{i,j})=\frac{{c}_{1,{r}_{i},{p}_{j}}*({c}_{2,{r}_{i},{p}_{j}}+{c}_{4,{r}_{i},{p}_{j}}*(1+{y}_{{r}_{i},{p}_{j}}-{x}_{{r}_{i},{p}_{j}}+{u}_{{r}_{i},{p}_{j}}))}{{c}_{2,{r}_{i},{p}_{j}}+{c}_{4,{r}_{i},{p}_{j}}*(1+{y}_{{r}_{i},{p}_{j}}-{x}_{{r}_{i},{p}_{j}}+{u}_{{r}_{i},{p}_{j}})+{c}_{3,{r}_{i},{p}_{j}}}-\frac{{c}_{1,{r}_{i},{p}_{j}}*({c}_{2,{r}_{i},{p}_{j}} + {c}_{4,{r}_{i},{p}_{j}}*{u}_{{r}_{i},{p}_{j}})}{{c}_{2,{r}_{i},{p}_{j}}+{c}_{4,{r}_{i},{p}_{j}}*{u}_{{r}_{i},{p}_{j}}+{c}_{3,{r}_{i},{p}_{j}}}$$

is the skill improvement function. While function (16) appears slightly different than function (9), this is only because of the difference in the variables used to model the disjunctive and time-indexed models. Both Eqs. (9) and (16) represent a modified hyperbolic function that models the learning curves [31, 32]. Passing a schedule represented in time-indexed model form through function (1) results in an equal value as passing that same schedule in disjunctive model form through function (10).

Constraint (11) ensures that the start and end times of a patient training on a given robot are between $1$ and $G$, and the start time is not later than the end time. Constraints (12) and (13) are disjunctive constraints on robot use and ensure that two patients’ training activities requiring the same robot cannot overlap in time. Specifically, for any two patients ${p}_{a},{p}_{b}\in P$, the start time of patient ${p}_{b}$ on a given robot must be at least ${e}_{{r}_{i}}$ greater than the end time of patient ${p}_{a}$ on the same robot if patient ${p}_{a}$ precedes ${p}_{b}$. This requirement accounts for the time, i.e., ${e}_{{r}_{i}}$, taken for patient ${p}_{a}$ to exit the robot. This is analogous to constraint (5) in the time-indexed model. The Boolean variable ${a}_{{r}_{i},{p}_{a},{p}_{b}}$ is introduced to indicate the precedence of two patients ${p}_{a}$ and ${p}_{b}$ on robot ${r}_{i}$. $V$ is a sufficiently large multiplier [35] that ensures that either (12) or (13), but not both, will hold depending on the precedence indicated by ${a}_{{r}_{i},{p}_{a},{p}_{b}}$. Note that the two constraints are taken into account only if both patient ${p}_{a}$ and ${p}_{b}$ use the robot $i$ during the training session, i.e., ${z}_{{r}_{i},{p}_{a}}= {z}_{{r}_{i},{p}_{b}}=1$. For any two patients ${p}_{a}$ and ${p}_{b}$ who do not both use robot ${r}_{i}$ at any time during the training session, i.e., ${z}_{{r}_{i},{p}_{a}}=0$ or ${z}_{{r}_{i},{p}_{b}}=0$, constraints (12) and (13) are always satisfied. Similarly, constraints (14) and (15) are disjunctive constraints on the schedule of a given patient that ensure that the start time of patient ${p}_{j}$ on robot ${r}_{b}$ must be at least ${e}_{{r}_{i}}$ greater than the end time of patient ${p}_{j}$ on robot ${r}_{a}$ if patient ${p}_{j}$ uses robot ${r}_{a}$ before robot ${r}_{b}$. The two constraints hold only if both robot ${r}_{a}$ and ${r}_{b}$ are used by patient ${p}_{j}$ during the training session, i.e., ${z}_{{r}_{a},{p}_{j}}={z}_{{r}_{b},{p}_{j}}=1$.

#### Simplifications for our study

While Eqs. (9) and (16) allow general representations of possible skill curves, our specific study held several variables constant for simplicity. ${c}_{1,{r}_{i},{p}_{j}}$ represents the maximum skill value that can be achieved with an infinite amount of training (e.g., maximum score on a clinical assessment scale); this was set to 100 for every skill. ${c}_{4,{r}_{i},{p}_{j}}$ determines how many “units” of training a patient gains in a skill when training that skill for one time step. This could be used to represent, e.g., more or less efficient robots for the same skill, but was not considered critical for the current study, and ${c}_{4,{r}_{i},{p}_{j}}$ was thus set to 1. Finally, we focused only on scenarios where patients have not previously trained with the robots, and ${u}_{{r}_{i},{p}_{j}}$ was thus set to 1. The skill function for the time-indexed model, Eq. (9), thus simplified to:

17 $${f(x}_{{r}_{i},{p}_{j},t},{d}_{{r}_{i},{p}_{j}})=\frac{100*({c}_{2,{r}_{i},{p}_{j}} + {d}_{{r}_{i},{p}_{j}}*{x}_{{r}_{i},{p}_{j},t}+1)}{{c}_{2,{r}_{i},{p}_{j}}+{d}_{{r}_{i},{p}_{j}}*{x}_{{r}_{i},{p}_{j},t}+1+{c}_{3,{r}_{i},{p}_{j}}} -\frac{100*{(c}_{2,{r}_{i},{p}_{j}}+1)}{{c}_{2,{r}_{i},{p}_{j}}+1+{c}_{3,{r}_{i},{p}_{j}}}$$

and Eq. (16) thus simplified to:

18 $${f(x}_{{r}_{i},{p}_{j}},{y}_{{r}_{i},{p}_{j}})=\frac{100*({c}_{2,{r}_{i},{p}_{j}}+2+{y}_{{r}_{i},{p}_{j}}-{x}_{{r}_{i},{p}_{j}})}{{c}_{2,{r}_{i},{p}_{j}}+2+{y}_{{r}_{i},{p}_{j}}-{x}_{{r}_{i},{p}_{j}}+{c}_{3,{r}_{i},{p}_{j}}}-\frac{100*{(c}_{2,{r}_{i},{p}_{j}}+1)}{{c}_{2,{r}_{i},{p}_{j}}+1+{c}_{3,{r}_{i},{p}_{j}}}$$

### Optimization algorithms

The Branch-And-Reduce Optimization Navigator (BARON) optimizer (The Optimization Firm LLC, USA) [36] was applied to both time-indexed and disjunctive models. BARON was chosen due to its ability to find global solutions to nonlinear and mixed-integer nonlinear problems. As the name implies, BARON specifically uses branch-and-bound optimization that always finds the global optimum under specific conditions (e.g., having a finite lower and upper bound on the nonlinear constraints, having enough optimization iterations to complete the search). Additionally, the OPTI toolbox for MATLAB 2021a (MathWorks, USA) was used to interface with the BARON optimizer, and IBM’s ILOG CPLEX optimization studio [37] was used to increase the effectiveness of the BARON optimizer. The OPTI toolbox can accept constraints in both linear and nonlinear format. Initially, we wrote all constraints in nonlinear format; for the final evaluation, linear constraints were written in linear format since this (on average) reduced optimization duration.

While the BARON optimizer is guaranteed to find a solution close to the global maximum, this is only if it has enough time to do so. As the number of optimization iterations is always limited, the optimizer may be unable to find the optimal schedule within that limit. Therefore, the optimization was combined with an algorithm that scans the schedule in each optimization iteration for obvious weaknesses and removes them. Specifically, the algorithm looks for situations where a patient is idle and scheduled to be assigned to a robot in later time steps, but that robot is already available earlier; in such situations, the algorithm changes the schedule so the patient begins training on that robot as soon as it is available. A similar process is applied to the end time: if a patient is scheduled to exit a robot but the robot and patient would then both be idle, the training time on the robot is extended. Despite this algorithm, repeated optimization of the same scenario may still lead to slightly different results due to the finite number of optimization iterations.

### Evaluation methodology

Our optimization strategy aims to maximize the total amount of skill gained during a session: the difference between the skill value at the end and the start of the session, summed across all patients and skills. This total skill gain depends on the patients’ initial skill level and their skill curves (modeled with Eqs. 17 and 18 for each patient-robot pairing). The skill curves have diminishing returns, similar to real-world situations where it becomes harder to improve a skill the more a patient has trained it [31, 32]. To evaluate the effectiveness of the optimization strategy, we first evaluated multiple scenarios where all patients have the same skill curve for all skills (Equal skill curves) and then multiple scenarios where the patients have different skill curves (Different skill curves). Finally, to evaluate the computational cost of the optimization, we measured how optimization duration depends on the number of patients, robots, and time steps (Effect of patients, robots and time steps on optimization duration).

#### Equal skill curves

In this situation, all skill curves of all patients are described using Eqs. (17) and (18) and the parameter values are ${c}_{3,{r}_{i},{p}_{j}}$ = 10, ${c}_{2,{r}_{i},{p}_{j}}$ = 1 and ${c}_{4,{r}_{i},{p}_{j}}$= 1 for each patient and skill. Three possible combinations of patients and robots were tested:

- 5 patients and 5 robots,
- 6 patients and 5 robots,
- 5 patients and 7 robots.

Each of these was tested in three time variants:

- 7 time steps total, robots have no exit time,
- 12 time steps total, robots have no exit time,
- 12 time steps total, robots have an exit time of 1 time step.

There was thus a total of 9 evaluation scenarios with equal skill curves.

In the last variant (1-step exit time), a patient must wait (remain idle) for one time step after training on a robot before they can be assigned to a new robot (${e}_{{r}_{i}}$= 1 $\forall {r}_{i}\in R$). Furthermore, no other patient can train with that robot until the previous patient has completed the idle period. Such exit times were not evaluated with a 7-time-step duration since the short duration would strongly favor not switching robots. The difference in computational complexity between 7 and 12 time steps was expected in advance to be greater for the time-indexed model since that model’s parameter count scales proportionally higher with respect to time while the disjunctive model’s does not.

For both models, the optimization was allowed to run for 1000 iterations. Additionally, two ‘baseline’ approaches were evaluated for each scenario:

- Best robot only: Each patient was assigned to a single robot for the entire session. This was selected as the robot that would result in the highest individual skill gain over the session for that patient. In case of conflicts (two patients would get highest gain from same robot), the robot was assigned to the patient who would receive a greater gain, and the other patient was assigned to their “second-best” robot.
- Switch halfway: Each patient was assigned to one robot for the first half of the session and a second robot for the second half of the session. These were again selected as the two robots that would result in the two highest individual skill gains over the session for that patient, and conflicts between patients were resolved similarly to the previous case. As the skill curves have diminishing returns, this was expected to lead to higher overall skill gain than not switching robots and is similar to a recent robotic gym paper where patients switched midway through the session [6].

As a basic statistical test, a one-way repeated-measures analysis of variance (ANOVA) with Holm-Sidak post-hoc tests was calculated with four conditions (disjunctive, time-indexed, best robot only, switch halfway) and nine samples per condition (the nine evaluation scenarios). A priori, both disjunctive and time-indexed models were expected to outperform both baseline schedules: optimization should yield a superior result unless the naïve schedules are already optimal. No significant difference was expected between disjunctive and time-indexed schedules; given infinite optimization iterations, both models should converge to the same schedule. It should be noted that the ANOVA independence assumption is violated since the “best robot only” result is the same in the “12 time steps” and “12 time steps and exit time” scenarios; we considered this acceptable since the ANOVA is simply a quick validation of the results rather than the primary outcome measure.

#### Different skill curves

Realistically, patients have different initial skill levels before training (e.g., different impairment levels after neurological injury) and learn skills at different rates. To represent these differences, every patient-robot pairing was assigned a different skill curve by varying variables ${c}_{2,i,j}$ and ${c}_{3,i,j}$ in Eqs. (17) and (18). These variables affect both the rate of growth and initial skill value.

42 different skill curves with different values of ${c}_{2,{r}_{i},{p}_{j}}$ and ${c}_{3,{r}_{i},{p}_{j}}$ were created and randomly distributed to patients and robots in a group. ${c}_{2,{r}_{i},{p}_{j}}$ ranged from 0.01 to 100 while ${c}_{3,{r}_{i},{p}_{j}}$ ranged from 5 to 1000. This was done three times to create three ‘groups’ of patients with different skill curves, allowing us to determine whether the system could consistently create a good schedule that was not dependent on a very specific set of skill distributions. For each of the three groups, the same 9 scenarios as in the previous subsection were simulated, and the two baseline approaches were evaluated as well. Again, optimizations ran for 1000 iterations.

As a basic statistical test, a two-way mixed ANOVA was conducted with one within-subject factor (schedule: disjunctive, time-indexed, best robot only, switch halfway), one between-subjects factor (group: 1–3) and nine samples per bin (nine evaluation scenarios). Holm-Sidak post-hoc tests were used to compare schedules, and effect size was reported as partial eta-squared. Significant differences between schedules were expected a priori since optimization should yield a superior result unless the naïve schedules are already optimal. Thus, the ANOVA serves as a quick check of the optimization rather than as the primary outcome.

#### Effect of patients, robots and time steps on optimization duration

The optimization duration is expected to increase as the number of patients, robots, and time steps increases. To determine the effect of each of these parameters, we applied both disjunctive and time-indexed models to the “equal skill curves” scenario, varied the three parameters, and measured the optimization duration. The following variations were tested:

- At 5 patients and 5 robots, test 1, 2, 3, 4, 5, and 12 time steps,
- At 5 robots and 5 time steps, test 1, 2, 3, 4 and 5 patients,
- At 5 patients and 5 time steps, test 1, 2, 3, 4, and 5 robots.

All optimizations were run on a personal computer with an 8-core 3600-MHz Ryzen 7 3700X central processing unit (AMD, Santa Clara, CA).

## Results

### Equal skill curves

The returned score function value represents the total skill gain: the difference between the skill value at the end and the start of the session, summed across all patients and skills. Table 1 shows total skill gain obtained with the different schedule types for the nine scenarios with equal skill curves.

**Table 1:** Total skill gain with different schedule types for the nine evaluation scenarios when all skill curves were the same

Scenario (PxRxT)	Disjunctive	Time-indexed	Best robot	Switch halfway
5P 5R 7 T	206.6	208.8	153.5	187.5
5P 5R 12 T	335.2	314.1	208.3	277.8
5P 5R 12 T with exit time	270.8	266.1	208.3	261.4
6P 5R 7 T	216.3	219.8	153.5	187.5
6P 5R 12 T	341.3	339.4	208.3	277.8
6P 5R 12 T with exit time	270.8	264.3	208.3	261.4
5P 7R 7 T	222.5	221.8	153.5	187.5
5P 7R 12 T	348.5	343.3	208.3	277.8
5P 7R 12 T with exit time	270.2	264.7	208.3	261.4

P represents the number of patients, R represents the number of robots, and T represents the training duration

The one-way repeated-measures ANOVA was significant (p < 0.001), and post-hoc tests found that both disjunctive and time-indexed schedules resulted in higher total skill gain than the “best robot” and “switch halfway” schedules (p < 0.01 in all cases). Switching halfway resulted in higher total skill gain than the “best robot” schedule (p < 0.001), but there was no significant difference between disjunctive and time-indexed schedules.

### Different skill curves

Tables 2, 3, and 4 show the total skill gains obtained with different schedule types for the nine scenarios with different skill curves. Each table corresponds to one of the three patient groups (skill curve assignments).

**Table 2:** Total skill gain after training for group 1 with the different schedule types for the nine different scenarios

Scenario (PxRxT)	Disjunctive	Time-indexed	Best robot	Switch halfway
5P 5R 7 T	110.5	107.6	83.9	100.0
5P 5R 12 T	163.2	147.7	115.5	134.5
5P 5R 12 T with exit time	148.2	139.5	115.5	128.9
6P 5R 7 T	119.0	114.7	95.3	109.6
6P 5R 12 T	176.0	156.5	131.1	147.0
6P 5R 12 T with exit time	158.9	151.1	131.1	139.3
5P 7R 7 T	184.0	166.7	142.7	174.0
5P 7R 12 T	254.6	232.3	187.3	219.5
5P 7R 12 T with exit time	231.8	221.6	187.3	211.1

P represents the number of patients, R represents the number of robots, and T represents the training duration

**Table 3:** Total skill gain after training for group 2 with the different schedule types for the nine different scenarios

Scenario (PxRxT)	Disjunctive	Time-indexed	Best robot	Switch halfway
5P 5R 7 T	189.4	184.2	136.9	177.9
5P 5R 12 T	254.0	234.6	173.4	234.1
5P 5R 12 T with exit time	241.0	230.8	173.4	224.0
6P 5R 7 T	199.2	196.7	145.1	186.4
6P 5R 12 T	267.9	245.9	186.4	241.4
6P 5R 12 T with exit time	253.8	234.8	186.4	231.2
5P 7R 7 T	211.2	206.8	137.8	197.0
5P 7R 12 T	280.8	266.7	175.0	261.4
5P 7R 12 T with exit time	261.9	255.0	175.0	248.2

P represents the number of patients, R represents the number of robots, and T represents the training duration

**Table 4:** Total skill gain after training for group 3 with the different schedule types for the nine different scenarios

Scenario (PxRxT)	Disjunctive	Time-indexed	Best robot	Switch halfway
5P 5R 7 T	135.8	134.4	102.3	118.3
5P 5R 12 T	193.2	183.0	132.2	171.7
5P 5R 12 T with exit time	177.2	175.2	132.2	162.0
6P 5R 7 T	169.5	163.6	138.4	147.9
6P 5R 12 T	230.0	212.4	171.7	208.2
6P 5R 12 T with exit time	215.0	209.2	171.7	198.2
5P 7R 7 T	173.9	173.3	137.2	170.1
5P 7R 12 T	244.1	234.8	175.2	225.1
5P 7R 12 T with exit time	227.5	205.3	175.2	214.8

P represents the number of patients, R represents the number of robots, and T represents the training duration

The ANOVA found a significant main effect of schedule (p < 0.001, partial eta-squared = 0.92) and a significant interaction effect of schedule × group (p < 0.001, partial eta-squared = 0.56). In post-hoc tests, both disjunctive and time-indexed schedules resulted in higher total skill gain than both baseline schedules (p < 0.001 for all comparisons), the disjunctive schedule resulted in higher total skill gain than the time-indexed schedule (p < 0.001), and the “switch halfway” schedule resulted in higher total skill gain than the “best robot” schedule (p < 0.001).

**Fig. 1:**
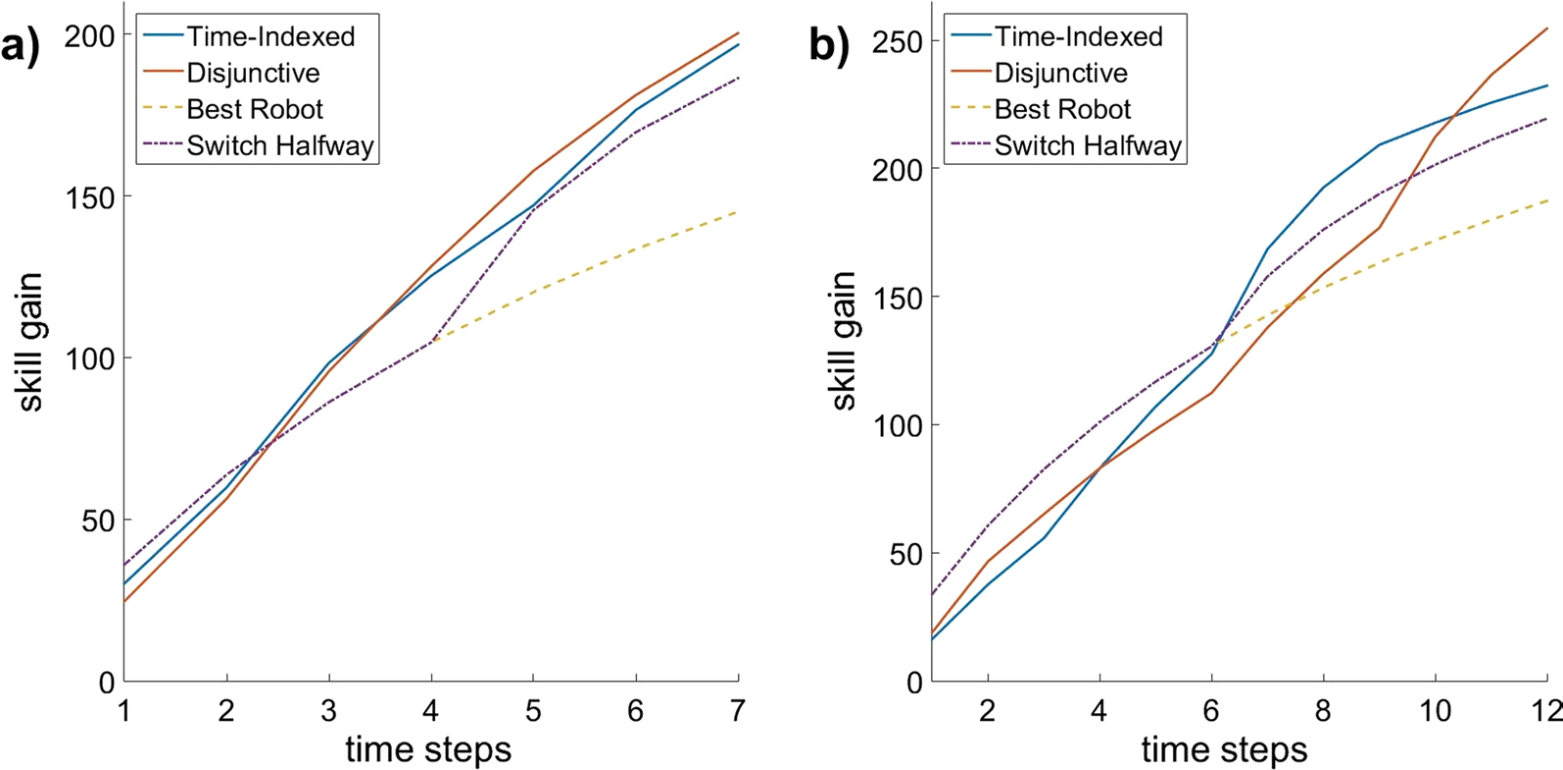
Two examples of total skill gain over time using four schedule types: disjunctive, time-indexed system, best robot, and switch halfway. Example **a** is for group 2 with 6 patients, 5 robots, and 7 time steps. Example **b** is for group 1 with 5 patients, 7 robots, and 12 time steps

**Fig. 2:**
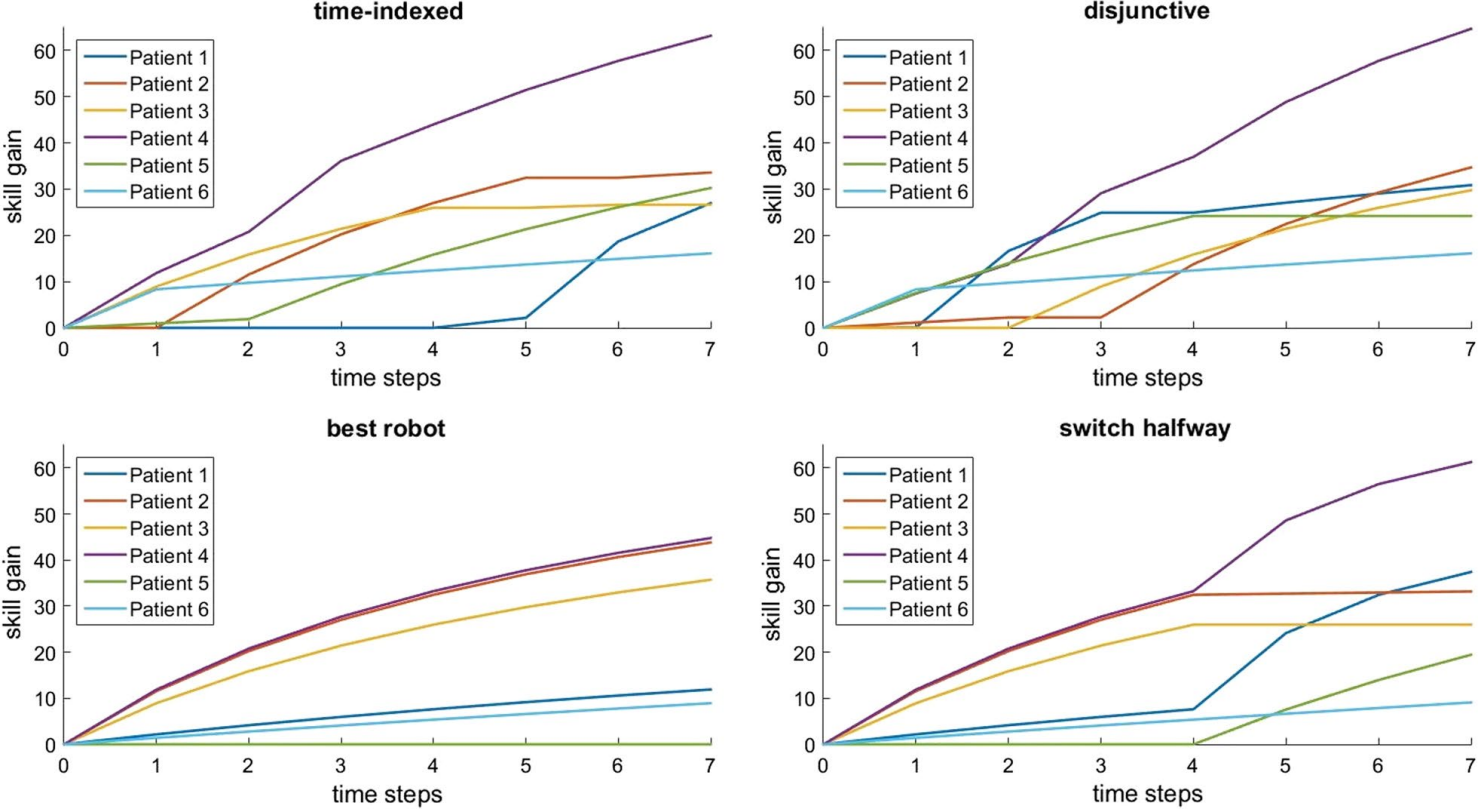
Total skill gain for each patient over time in group 2 with 6 patients, 5 robots, and 7 time steps. The subplots represent different scheduling approaches

**Fig. 3:**
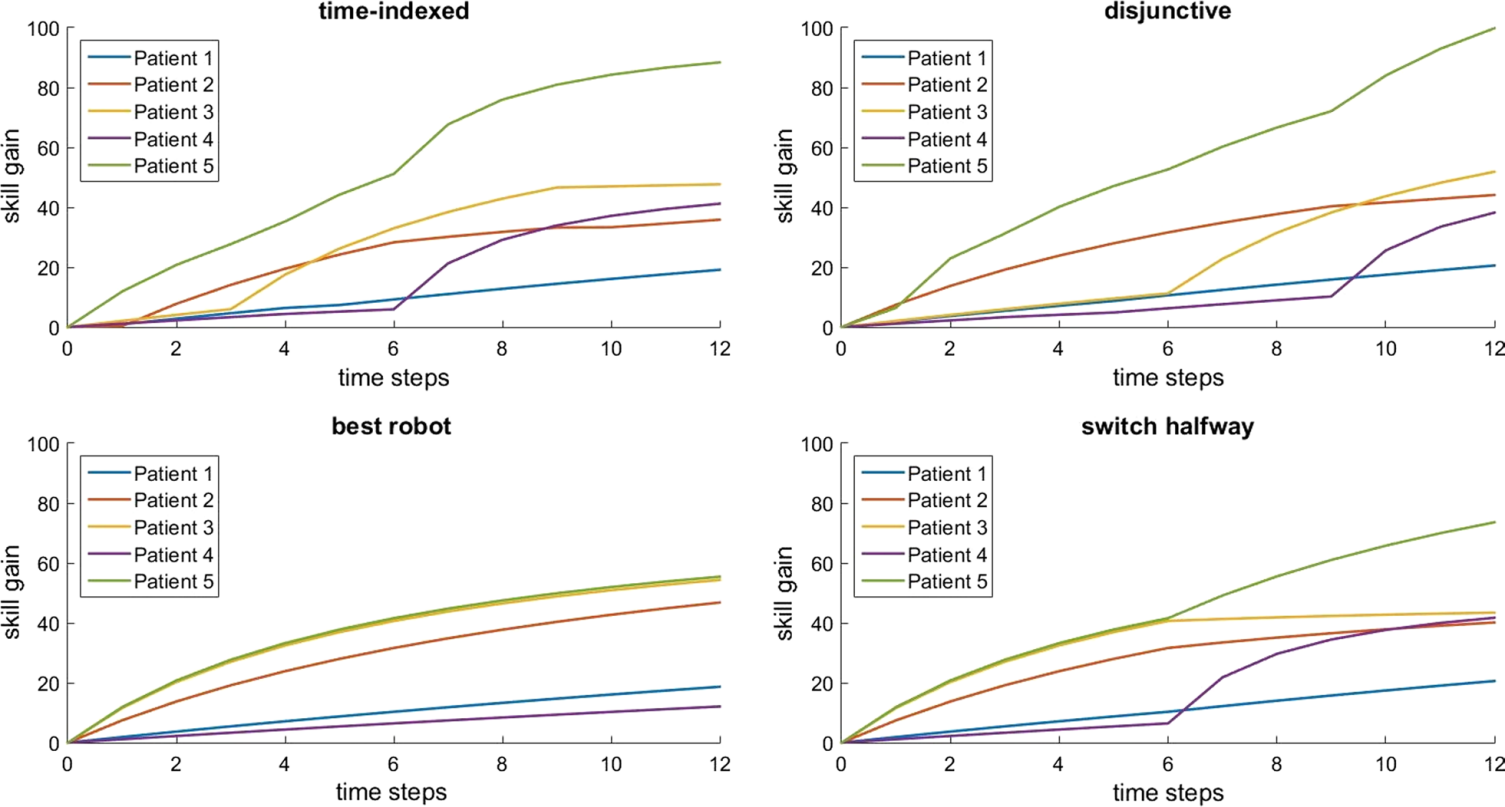
Total skill gain for each patient over time in group 1 with 5 patients, 7 robots, and 12 time steps. The subplots represent different scheduling approaches

Figure 1 shows a visual representation of the total skill gain over time (rather than only at the end of the session) using the four schedule types (disjunctive, time-indexed, best robot, switch halfway) for two representative examples: (a) 6 patients, 5 robots and 7 time steps, and (b) 5 patients, 7 robots and 12 time steps (right). Figures 2 and 3 show the total skill gain over time in the same two examples and with the same schedule types, but separately for each individual patient rather than as a group.

### Effect of patients, robots and time steps on optimization duration

As mentioned, this evaluation was done with the “equal skill curves” scenario. The following results were obtained for the disjunctive model:

- When varying the number of time steps with 5 patients and 5 robots, the optimization duration is 0.3 s for 1 time step, 11.9 s for 2 steps, 17.7 s for 3 steps, 61.3 s for 4 steps, 1201 s for 5 steps, and 2056s for 12 steps.
- When varying the number of patients with 5 robots and 5 time steps, the optimization duration is 0.3 s for 1 patient, 3.6 s for 2 patients, 240 s for 3 patients, 273 s for 4 patients, and 1201 s for 5 patients.
- When varying the number of robots with 5 patients and 5 time steps, the optimization duration is 0.3 s for 1 robot, 33.9 s for 2 robots, 51.1 s for 3 robots, 69.8 s for 4 robots, and 1201 for 5 robots.

The following results were obtained for the time-indexed model:

- When varying the number of time steps with 5 patients and 5 robots, the optimization duration is 0.1 s for 1 time step, 4.1 s for 2 steps, 23.8 s for 3 steps, 380 s for 4 steps, 1479 s for 5 steps, and 16,400 s for 12 steps.
- When varying the number of patients with 5 robots and 5 time steps, the optimization duration is 0.3 s for 1 patient, 24 s for 2 patients, 717 s for 3 patients, 800 s for 4 patients, and 1479 s for 5 patients.
- When varying the number of robots with 5 patients and 5 time steps, the optimization duration is 0.3 s for 1 robot, 51 s for 2 robots, 287 s for 3 robots, 299 s for 4 robots, and 1479 s for 5 robots.

These durations vary somewhat whenever the optimization is re-run, but we consider an approximate time to be sufficient for illustration.

## Discussion

### Equal skill curves

The time-indexed and disjunctive systems significantly outperformed the baseline schedules, as indicated by the repeated-measures ANOVA and Table 1. In all scenarios, both models were always better than the “best robot” schedule. While there was no statistically significant difference in total skill gain between time-indexed and disjunctive systems, there were some nonsignificant differences. Most prominently, there were a few scenarios with 1-step exit times where the disjunctive system outperformed the “switch halfway” schedule but the time-indexed system did not (Table 1).

Both time-indexed and disjunctive systems should converge to the same schedule given infinite optimization iterations. The outcome differences are due to a finite number of optimization iterations, which gives an advantage to the simpler disjunctive model. In that model, start and stop times can be changed simply by changing integer values assigned to a patient-robot pairing. Conversely, in the time-indexed system, the starting time of a patient on a robot is determined by a binary variable. Thus, to change the time when a patient starts training on a robot, the system must toggle off one binary variable, toggle another one on, and possibly change the duration integer, resulting in an overall more difficult optimization process that requires more iterations. To verify that time-indexed and disjunctive systems would converge to the same schedule given infinite optimization iterations, we later applied both models to several simpler scenarios (3 patients, 3 robots, 12 time steps; 5 patients, 5 robots, 1–3 time steps) and ran optimizations until no changes occurred for 2000 iterations. In all cases, disjunctive and time-indexed models converged on the same schedule.

While the variables used in the time-indexed model make the optimization take longer, they do remove an inherent limitation of the disjunctive system. The disjunctive model only allows a patient to train on a robot once within a session. While this works for the constraints in this paper, it would not work in a less constrained situation where there is no limit on how often a patient can train on a robot. Conversely, the time-indexed model could be easily modified to remove this constraint.

### Different skill curves

When the skill curves were not equal, both disjunctive and time-indexed schedules significantly outperformed both baseline schedules. This was expected since optimization should be more effective than a naïve schedule. However, the difference in total skill gain between disjunctive and time-indexed schedules was now statistically significant, with the disjunctive schedule overall resulting in higher total skill gain. Additionally, the time-indexed system was worse than the “switch halfway” schedule in two scenarios while the disjunctive system always resulted in higher total skill gain than the “switch halfway” schedule. While disjunctive and time-indexed systems should converge to the same schedule given infinite iterations, the disjunctive system thus appears to be preferable given finite optimization durations that are likely to be seen in realistic robot gyms.

As Fig. 1 shows, the baseline schedules outperform disjunctive and time-indexed schedules in initial time steps, but the opposite becomes true in later time steps. This is due to diminishing returns in individual skill curves: as patients’ skill gain on a robot decreases with time spent on that robot, assigning a patient to a single robot (as in the baseline schedules) has high initial skill gains. Conversely, the optimized schedules can plan over the long term, sacrificing high initial gains for a higher total gain.

Figures 2 and 3 also show that, while disjunctive and time-indexed systems result in higher total skill gain than baseline schedules, not all patients benefit equally. For example, in Fig. 2, patient 5 has no skill gain at all in the “best robot” schedule; since there are more patients than robots, one patient must be neglected in the “best robot” schedule, and patient 5 benefits more from other schedules. Conversely, patient 3 exhibits the highest gain in the “switch halfway” schedule rather than in an optimized schedule. This is less of an issue in Fig. 3, where there are more robots than patients. Nonetheless, optimizing for highest total skill gain does not mean that all patients benefit equally, and we discuss the implications later.

Finally, the significant interaction effect of group x schedule indicates that the three groups had different skill gains, with group 2 on average having the highest gains. This does not, however, mean that the system performed better for group 2. As skill curve distributions are different between groups, some groups simply have more potential for skill gain. In the future, it would be beneficial to determine what factors determine the potential of optimization, allowing researchers to decide when optimization should be performed. For example, instead of only having three groups, a Monte Carlo simulation could be used to generate many different groups, and the impact of each parameter in Eqs. (9) and (16) could be evaluated. However, we believe that more complex scenarios should be created (Expanding the scenario simplifications) and optimization duration should be reduced (Optimization duration) before such detailed evaluations are conducted.

### Optimization duration

While disjunctive and time-indexed models can converge toward an optimal schedule regardless of problem complexity as long as specific conditions are met, “Effect of patients, robots and time steps on optimization duration” shows that more time and optimization iterations are generally needed to reach the optimum as the number of robots, patients or time steps increases. Due to its relative simplicity, the disjunctive system overall requires less time for optimization than the time-indexed system: for example, the most complex scenario required 2056 s with the disjunctive system but 16,400 s with the time-indexed system. The disjunctive system is thus again considered preferable for realistic situations where limited time is available for optimization.

In our evaluations, the number of iterations was set to 1000, but the schedule is not necessarily improved in every iteration. Generally, 3–6 meaningful ‘improvements’ to the schedule occurred over the course of these iterations; however, it is impossible to know beforehand when these improvements will occur. Thus, increasing the iteration cap above 1000 may result in slightly better outcomes at the cost of more optimization time. Conversely, reducing the iteration cap would reduce optimization time but may have either a minimal or critical effect on the final schedule.

The increase in optimization time as the number of patients, robots, or time steps increases is a byproduct of the problem being an MINLP. As MINLPs are NP-hard problems, an optimal solution cannot be found in polynomial time unless P = NP [38, 39]. The optimization duration thus increases exponentially with problem size and can become impractical if the problem is too complex, as seen in “Effect of patients, robots and time steps on optimization duration”. While there is no way to avoid exponential increases in optimization duration within the current framework, some steps can nonetheless be taken to speed up optimization. For example, in the current study, the BARON optimizer created all schedules with no initial guess. It would be possible to instead begin with an initial schedule (e.g., one created by a therapist based on a quick evaluation of the patients) and have the system try to improve upon it.

As another alternative within the current framework, the number of time steps can be varied by the person supervising the optimization. Increasing the number of time steps increases the session duration or improves schedule granularity (e.g., patients being able to switch robots every 5 min vs. every 10 min), but also exponentially increases optimization duration. Furthermore, the optimal schedule for a 7-time-step scenario is not necessarily the same as the optimal schedule for the first 7 steps of a 12-step scenario—the system may make different decisions in the first 7 steps if it ‘knows’ that more steps are available later. Thus, in a real-world robotic gym, the optimization supervisor could choose a preferable tradeoff between schedule duration/granularity, schedule optimality (related to number of optimization iterations), and computational cost.

Finally, to overcome the limitations of MINLP, we have begun work on a different patient-robot assignment framework that does not use an optimizer. Instead, it uses a neural network trained to predict skill growth. Preliminary results have shown that the neural network approach drastically reduces the time needed to create a schedule, and we are currently combining it with a more complex scenario (Expanding the scenario simplifications).

### Different optimization goals

In the current study, the optimization goal was always the same: maximizing total skill gain across all patients and skills. While this can be desirable in many cases, it may also have downsides in real-world situations. For example, it may compromise the rehabilitation of patients who are considered “less promising”, reducing their quality of life post-rehabilitation. Even if no patient is neglected, the same absolute gain does not always have the same practical meaning. For example, the Fugl-Meyer Assessment upper limb score can range from 0 to 66 [28], but improving it from 0 to 6 may have different implications for the patient than improving it from 60 to 66.

To address this issue, future studies could optimize different objective functions. As a preliminary follow-up, we have implemented two alternative objective functions for the time-indexed model that aim to distribute skill gains more evenly among patients. First, we modified objective function (17) to include a penalty element corresponding to the variance in skill gain among patients, thus ensuring that all patients improve to a similar degree. This resulted in the following objective function:

19 $${f(x}_{{r}_{i},{p}_{j},t},{d}_{{r}_{i},{p}_{j}})=\frac{100*\left({c}_{2,{r}_{i},{p}_{j}} + {d}_{{r}_{i},{p}_{j}}*{x}_{{r}_{i},{p}_{j},t}+1\right)}{{c}_{2,{r}_{i},{p}_{j}}+{d}_{{r}_{i},{p}_{j}}*{x}_{{r}_{i},{p}_{j},t}+1+{c}_{3,{r}_{i},{p}_{j}}} -\frac{100*{(c}_{2,{r}_{i},{p}_{j}}+1)}{{c}_{2,{r}_{i},{p}_{j}}+1+{c}_{3,{r}_{i},{p}_{j}}} -m *variance(\frac{100*({c}_{2,{r}_{i},{p}_{j}} + {d}_{{r}_{i},{p}_{j}}*{x}_{{r}_{i},{p}_{j},t}+1)}{{c}_{2,{r}_{i},{p}_{j}}+{d}_{{r}_{i},{p}_{j}}*{x}_{{r}_{i},{p}_{j},t}+1+{c}_{3,{r}_{i},{p}_{j}}} -\frac{100*{(c}_{2,{r}_{i},{p}_{j}}+1)}{{c}_{2,{r}_{i},{p}_{j}}+1+{c}_{3,{r}_{i},{p}_{j}}})$$

where m is a penalty coefficient.

Second, we implemented an objective function that takes each patient’s maximum possible skill gain into account. First, the optimization is run with each patient individually (number of patients = 1) using objective function (17) to obtain that patient’s maximum possible skill gain if they are the only patient in the gym. The optimization is then run for all patients together with a new objective function that aims to optimize total skill gain relative to each patient’s individual maximum possible gain:

20 $$\mathrm{max}\sum_{j=1}^{M}(\sum_{i=1}^{N}\sum_{t=1}^{H}{f(x}_{{r}_{i},{p}_{j},t},{d}_{{r}_{i},{p}_{j}})-{m}_{{p}_{j}}*(1-\frac{\sum_{{r}_{i}=1}^{N}\sum_{t=1}^{H}{f(x}_{{r}_{i},{p}_{j},t},{d}_{{r}_{i},{p}_{j}})}{maximum \;theoretical\; training\; gain\; for\;{p}_{j} }))$$

where ${f(x}_{{r}_{i},{p}_{j},t},{d}_{{r}_{i},{p}_{j}})$ is Eq. (17) and m ${p}_{j}$ is a penalty coefficient for patient ${p}_{j}$. This coefficient allows the priority of each patient to be modified as desired.

**Fig. 4:**
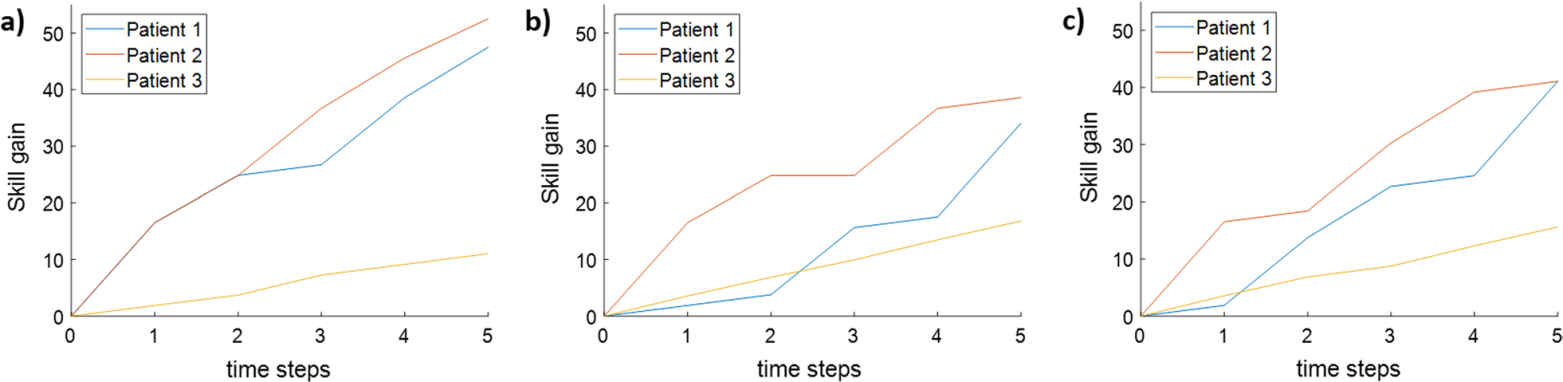
A preliminary comparison of total skill gain for each patient over time using **a** the original time-indexed system, **b** a modified system whose objective function includes a penalty corresponding to variance in skill gain among patients, and **c** a modified system that aims to optimize patients’ skill gains relative to their individual maximum possible gains. All examples are for 3 patients, 5 robots, 5 time steps, different skill curves

We then applied the time-indexed model to a simple scenario (3 patients, 5 robots, 5 time steps, different skill curves) using the original objective function (17) and new functions (19) and (20). This resulted in the example skill gains shown in Fig. 4. The example shows that the two new functions lead to more similar skill gains among patients, resulting in a benefit for patient 3 but losses for the other two patients. These functions therefore also have drawbacks; for example, they may unfairly slow down patients with more “potential”. The most appropriate optimization goal may be dependent on the situation: for example, groups of patients with similar impairment levels or similar potential for improvement may benefit from a different optimization goal than more heterogeneous groups.

### Expanding the scenario simplifications

As mentioned in the Methods, two scenario simplifications are quite severe. First, we assumed that each patient’s current skill levels are known perfectly and that skill improvement curves are deterministic. Realistically, significant uncertainty is involved in skill improvement and assessment, and would need to be considered (Stochastic modeling). Second, we assumed that skill improvement depends only on time spent training the skill. Realistically, improvement is influenced by multiple factors related to the patient and rehabilitation environment, which would also need to be considered (Robot and patient characteristics). After discussing these possible expansions, we present our long-term view of how dynamic patient-robot assignment algorithms would realistically be used (Long-term vision).

#### Stochastic modeling

Multiple sources of uncertainty could be included in a simulated robot gym. For example, patient skill level realistically would not be observable directly, and would need to be estimated from measurements such as task performance (i.e., success rate in the exercise) as well as the amount, quality (e.g., smoothness) and intensity of movement [21–24]. This could be simulated by representing patient skill level as a hidden state from which observable outputs are generated via a model that describes the probability distribution of output values in a given hidden state. Second, patient skill improvement over time would be a stochastic process that could be simulated using a probabilistic model such as Markov process or Gaussian process. Patient skill forgetting and spontaneous recovery could be modeled as random changes between consecutive sessions, and standardized clinical tests could be modeled as an accurate skill estimate that can only be done between sessions. Finally, patients arriving and leaving one by one could be modeled as different start/stop times for each patient within a session that may or may not be known to the optimization algorithm in advance.

Once these uncertainties have been incorporated, the optimization algorithm would need to be executed dynamically after each time step. In the current “static” formulation, the algorithm determines the entire session schedule as an open-loop solution at the beginning of the session since patient skill improvement is perfectly predictable given the deterministic model. However, a schedule determined ahead of time cannot be effective in the presence of uncertainties. In a dynamic schedule, the algorithm would incorporate new information (e.g., actual measurements, unexpected patient arrival/departure) after each time step and make patient-robot assignment decisions for the next time step.

#### Robot and patient characteristics

Multiple characteristics of robots and patients could be considered in a robotic gym. For example, rehabilitation robots commonly feature selectable difficulty levels [13, 14] and control strategies (e.g., assistive vs challenge-based [5]). These could be incorporated as a two-stage patient-robot assignment algorithm: first assign a patient to a robot, then choose the robot’s settings. In an expanded model where patient ‘hidden’ skill is observable via performance and other outputs (see previous subsection), these settings may influence both skill improvement and observable outputs. For example, if difficulty is too low, patients may exhibit high performance but low skill improvement. Challenge-based strategies may lead to lower skill improvement in unskilled patients than assistive strategies, but higher improvement in more skilled patients. Finally, some robots may include generalization of skill acquisition to other skills: a large gain in the primary skill (as in the current study) as well as smaller gains in other skills.

Additionally, patient motivation and engagement have a significant effect on immediate performance [7–9, 11] and long-term functional gains [40]. This could be modeled by having unmotivated patients exhibit worse task performance and lower skill improvement. Motivation could also be modified by events within a session itself. For example, patients may dislike specific robots (and lose motivation if assigned to them) or switching robots too frequently. An excessive difficulty setting may decrease motivation, and not assigning a patient to any robot may decrease it as well (due to patient perception of being neglected). Such a patient model could also include fatigue as a related factor. For example, fatigue may increase as the patient exercises (especially at high difficulty settings), leading to decreased motivation. Fatigue could be decreased by not assigning a patient to any robot for a time step. However, if motivation decreases below a threshold, the patient may even leave the session unexpectedly.

Finally, the type and degree of patient impairment could be modeled as having an impact not only on initial patient skill levels, but also skill curves. For example, while we currently modeled all patients as having skill curves with diminishing returns, we could instead model a mix of curves: some with diminishing returns and some with, e.g., increasing returns [31, 32].

#### Long-term vision

As mentioned in “Stochastic modeling”, optimization algorithms would likely need to be executed dynamically after each time step due to the presence of uncertainties. Additionally, computational cost increases with the number of time steps to be optimized (Effect of patients, robots and time steps on optimization duration). Thus, we believe that, in the long term, robot gyms will not have a fully fixed session schedule. Instead, before the session, the optimization algorithm will create a tentative schedule for the first few time steps based on patients’ medical files and data from previous sessions. The therapist will then move around the gym assisting individual patients while the robot gym software monitors all patients as a group. The software will dynamically re-optimize the schedule for the next few time steps as new data become available and will provide suggestions to the therapist when it believes that a patient should be moved to a different robot. As the computational cost for optimizing 1–3 time steps is relatively low, this can be done during the session itself, allowing a therapist to focus on helping individual patients without having to think about gym scheduling.

### Learning from human experts

Finally, our study focused on purely computer-driven optimization without any human knowledge. In the future, an alternative approach could be to learn by demonstration from a human expert. For example, a therapist could manually assign patients to robots during a session, and their decisions could be recorded together with task performance metrics and robot sensor data. Supervised machine learning could then be used to train a patient-robot assignment policy to mimic the therapist’s decisions. For example, a related work [41] presented a framework for learning a set of heuristics from human demonstration for resource allocation and scheduling in a patient care scenario. This would require an entirely different class of algorithms but may represent a more practical and realistic implementation approach.

The main challenge with such an approach is that suitable datasets are currently unavailable and would need to be obtained in a well-equipped robot gym that is currently only accessible to a few rehabilitation facilities. As an initial step, a dataset could be generated by human interaction with a simulation. For example, a modified version of the simulation could be designed to present a human expert with simulated patients’ skill levels (or measurable variables as described in “Stochastic modeling”) after each time step, and the expert could then make manual patient-robot assignment choices after each time step. While this would still suffer from similar simplifications as the current scenario, it would allow machine learning algorithms to be evaluated on a simulated dataset that nonetheless involves a real human expert.

## Conclusions

Our study presented a simplified model of a robotic rehabilitation gym, where multiple patients train with multiple robots to learn different skills. Time-indexed and disjunctive models were used to optimize total skill gain across all patients and skills within a training session. Both optimization models significantly outperformed two baseline schedule types: having each patient stay on a single robot throughout the session and having patients switch robots halfway through the session. The disjunctive model resulted in higher total skill gain and required less optimization time than the time-indexed model in the given scenarios. Though our simulation study involved unrealistically simple scenarios, it thus demonstrated that intelligently moving patients between rehabilitation robots can improve skill acquisition in a multi-patient multi-robot environment. Finally, we discussed how these simplifications could be expanded on in the future.

While robotic rehabilitation gyms have not yet become commonplace in clinical practice, prototypes of them already exist and are likely to become increasingly popular as the price of rehabilitation robots decreases. Our study thus presents a way to use automated decision-making and decision support to support chronically overworked physical and occupational therapists, allowing them to effectively supervise a larger number of patients undergoing rehabilitation. While numerous challenges would need to be solved before the envisioned system could be used in practice, it could in the long term allow more efficient delivery of technologically aided rehabilitation, and may be broadly applicable to other scenarios where groups of human work with groups of robots or virtual agents to learn skills.

## Data Availability

All code developed in the current study is publicly available on Zenodo at https://zenodo.org/record/7308921 (https://doi.org/10.5281/zenodo.7308921).

## Abbreviations

BARON: Branch-And-Reduce Optimization Navigator
MINLP: Mixed-integer nonlinear programming
ANOVA: Analysis of variance

## References

[1] Lo AC, Guarino PD, Richards LG, Haselkorn JK, Wittenberg GF, Federman DG (2010). Robot-assisted therapy for long-term upper-limb impairment after stroke. N Engl J Med.

[2] Klamroth-Marganska V, Blanco J, Campen K, Curt A, Dietz V, Ettlin T (2014). Three-dimensional, task-specific robot therapy of the arm after stroke: a multicentre, parallel-group randomised trial. Lancet Neurol.

[3] Aprile I, Germanotta M, Cruciani A, Loreti S, Pecchioli C, Cecchi F (2020). Upper limb robotic rehabilitation after stroke: a multicenter, randomized clinical trial. J Neurol Phys Ther.

[4] Fisher Bittmann M, Patton JL (2017). Forces that supplement visuomotor learning: a “sensory crossover” experiment. IEEE Trans Neural Syst Rehabil Eng.

[5] Marchal-Crespo L, Reinkensmeyer DJ. Review of control strategies for robotic movement training after neurologic injury. J Neuroeng Rehabil. 2009;6. 10.1186/1743-0003-6-20 PMC2710333 19531254

[6] Demofonti A, Carpino G, Zollo L, Johnson MJ (2021). Affordable robotics for upper limb stroke rehabilitation in developing countries: a systematic review. IEEE Trans Med Robot Bionic.

[7] Novak D, Nagle A, Keller U, Riener R (2014). Increasing motivation in robot-aided arm rehabilitation with competitive and cooperative gameplay. J Neuroeng Rehabil.

[8] Baur K, Schättin A, de Bruin ED, Riener R, Duarte JE, Wolf P. Trends in robot-assisted and virtual reality-assisted neuromuscular therapy: a systematic review of health-related multiplayer games. J Neuroeng Rehabil. 2018;15. 10.1186/s12984-018-0449-9 PMC6245892 30454009

[9] Pereira F, Bermúdez i Badia S, Jorge C, Cameirão MS (2021). The use of game modes to promote engagement and social involvement in multi-user serious games: a within-person randomized trial with stroke survivors. J Neuroeng Rehabil.

[10] Johnson MJ, Loureiro RCV, Harwin WS (2008). Collaborative tele-rehabilitation and robot-mediated therapy for stroke rehabilitation at home or clinic. Intell Serv Robot.

[11] Ballester BR, Bermúdez i Badia S, Verschure PFMJ (2012). Including social interaction in stroke VR-based motor rehabilitation enhances performance: a pilot study. Presence Teleoperators Virtual Environ..

[12] Batson JP, Kato Y, Shuster K, Patton JL, Reed KB, Tsuji T, et al. Haptic coupling in dyads improves motor learning in a simple force field. In: Proceedings of the 42nd Annual International Conference of the IEEE Engineering in Medicine and Biology Society. 2020. 10.1109/EMBC44109.2020.9176261 33019063

[13] Goršič M, Darzi A, Novak D. Comparison of two difficulty adaptation strategies for competitive arm rehabilitation exercises. In: Proceedings of the 2017 IEEE International Conference on Rehabilitation Robotics. London, UK; 2017. p. 640–5. 10.1109/ICORR.2017.8009320 PMC5669049 28813892

[14] Baur K, Wolf P, Riener R, Duarte J. Making neurorehabilitation fun: Multiplayer training via damping forces balancing differences in skill levels. In: Proceedings of the 2017 IEEE International Conference on Rehabilitation Robotics. 2017. 10.1109/ICORR.2017.8009359 28813931

[15] Goršič M, Cikajlo I, Goljar N, Novak D (2020). A multisession evaluation of a collaborative virtual environment for arm rehabilitation. Presence Virtual Augment Real.

[16] Wuennemann MJ, Mackenzie SW, Lane HP, Peltz AR, Ma X, Gerber LM (2020). Dose and staffing comparison study of upper limb device-assisted therapy. NeuroRehabilitation.

[17] Bustamante Valles K, Montes S, de Jesus Madrigal M, Burciaga A, Martínez ME, Johnson MJ (2016). Technology-assisted stroke rehabilitation in Mexico: a pilot randomized trial comparing traditional therapy to circuit training in a robot/technology-assisted therapy gym. J Neuroeng Rehabil.

[18] Jakob I, Kollreider A, Germanotta M, Benetti F, Cruciani A, Padua L (2018). Robotic and sensor technology for upper limb rehabilitation. Phys Med Rehabil.

[19] Aprile I, Pecchioli C, Loreti S, Cruciani A, Padua L, Germanotta M (2019). Improving the efficiency of robot-mediated rehabilitation by using a new organizational model: an observational feasibility study in an Italian rehabilitation center. Appl Sci.

[20] Bessler J, Prange-Lasonder GB, Schaake L, Saenz JF, Bidard C, Fassi I (2021). Safety assessment of rehabilitation robots: a review identifying safety skills and current knowledge gaps. Front Robot AI.

[21] Balasubramanian S, Colombo R, Sterpi I, Sanguineti V, Burdet E (2012). Robotic assessment of upper limb motor function after stroke. Am J Phys Med Rehabil.

[22] De Los R-G, Dimbwadyo-Terrer I, Trincado-Alonso F, Monasterio-Huelin F, Torricelli D, Gil-Agudo A (2014). Quantitative assessment based on kinematic measures of functional impairments during upper extremity movements: a review. Clin Biomech.

[23] Shirota C, Balasubramanian S, Melendez-Calderon A (2019). Technology-aided assessments of sensorimotor function: current use, barriers and future directions in the view of different stakeholders. J Neuroeng Rehabil.

[24] Tran V-D, Dario P, Mazzoleni S (2018). Kinematic measures for upper limb robot-assisted therapy following stroke and correlations with clinical outcome measures: a review. Med Eng Phys.

[25] Verhoeven FM, Newell KM (2018). Unifying practice schedules in the timescales of motor learning and performance. Hum Mov Sci.

[26] Lee JY, Oh Y, Kim SS, Scheidt RA, Schweighofer N (2016). Optimal schedules in multitask motor learning. Neural Comput.

[27] Carr JH, Stepherd RB, Nordholm L, Lynne D (1985). Investigation of a new motor assessment scale for stroke patients. Phys Ther.

[28] Fugl-Meyer AR, Jääskö L, Leyman I, Olsson S, Steglind S (1975). The post-stroke hemiplegic patient. 1. A method for evaluation of physical performance. Scand J Rehabil Med.

[29] Riener R, Dislaki E, Keller U, Koenig A, Van Hedel H, Nagle A (2013). Virtual reality aided training of combined arm and leg movements of children with CP. Stud Health Technol Inform.

[30] Mazzoleni S, Tran V-D, Dario P, Posteraro F (2018). Wrist robot-assisted rehabilitation treatment in subacute and chronic stroke patients: from distal-to-proximal motor recovery. IEEE Trans Neural Syst Rehabil Eng.

[31] Newell KM, Liu YT, Mayer-Kress G (2001). Time scales in motor learning and development. Psychol Rev.

[32] Mazur JE, Hastle R (1978). Learning as accumulation: a reexamination of the learning curve. Psychol Bull.

[33] Ku W-Y, Beck JC (2016). Mixed Integer Programming models for job shop scheduling: a computational analysis. Comput Oper Res.

[34] Kondili E, Pantelides CC, Sargent RWH. A general algorithm for scheduling batch operations. In: 3rd International Symposium on Process System Engineering. 1988. p. 62–75.

[35] Kanet JJ, Ahire SL, Gorman MF. Constraint programming for scheduling. In: Handbook of Scheduling, vol. 47. Chapman and Hall/CRC Press; 2004. p. 1–21.

[36] Tawarmalani M, Sahinidis NV (2005). A polyhedral branch-and-cut approach to global optimization. Math Program.

[37] CPLEX II. V12.8: User’s Manual for CPLEX. International Business Machines Corporation; 2017.

[38] Ali M, Qaisar S, Naeem M, Mumtaz S, Rodrigues JJPC (2020). Combinatorial resource allocation in D2D assisted heterogeneous relay networks. Futur Gener Comput Syst.

[39] Köppe M, Lee J, Leyffer S (2012). On the complexity of nonlinear mixed-integer optimization. Mixed integer nonlinear programming.

[40] Rapolienė J, Endzelytė E, Jasevičienė I, Savickas R (2018). Stroke patients motivation influence on the effectiveness of occupational therapy. Rehabil Res Pract.

[41] Gombolay M, Yang XJ, Hayes B, Seo N, Liu Z, Wadhwania S (2018). Robotic assistance in the coordination of patient care. Int J Rob Res.

